# Diurnal timing of physical activity in relation to obesity and diabetes in the German National Cohort (NAKO)

**DOI:** 10.1038/s41366-025-01721-9

**Published:** 2025-01-24

**Authors:** Michael J. Stein, Andrea Weber, Fabian Bamberg, Hansjörg Baurecht, Klaus Berger, Patricia Bohmann, Hermann Brenner, Julian Brummer, Marcus Dörr, Beate Fischer, Sylvia Gastell, Karin Halina Greiser, Volker Harth, Antje Hebestreit, Jana-Kristin Heise, Florian Herbolsheimer, Till Ittermann, André Karch, Thomas Keil, Alexander Kluttig, Lilian Krist, Karin B. Michels, Rafael Mikolajczyk, Matthias Nauck, Katharina Nimptsch, Nadia Obi, Tobias Pischon, Olga Pivovarova-Ramich, Tamara Schikowski, Börge Schmidt, Matthias B. Schulze, Karen Steindorf, Stephanie Zylla, Michael F. Leitzmann

**Affiliations:** 1 Department of Epidemiology and Preventive Medicine, University of Regensburg, Regensburg, Germany; 2 Department of Diagnostic and Interventional Radiology, Medical Center—University of Freiburg, Faculty of Medicine, University of Freiburg, Freiburg, Germany; 3 Institute of Epidemiology and Social Medicine, University of Münster, Münster, Germany; 4 Division of Clinical Epidemiology and Aging Research, German Cancer Research Center (DKFZ), Heidelberg, Germany; 5 Division of Physical Activity, Prevention and Cancer, German Cancer Research Center (DKFZ), Heidelberg, Germany; 6 Medical Faculty, Heidelberg University, Heidelberg, Germany; 7 Department of Internal Medicine B, University Medicine Greifswald, Greifswald, Germany; 8 German Centre for Cardiovascular Research (DZHK), partner site Greifswald, Greifswald, Germany; 9 Department of Molecular Metabolism and Precision Nutrition, German Institute of Human Nutrition Potsdam-Rehbruecke, Nuthetal, Germany; 10 Division of Cancer Epidemiology, German Cancer Research Center (DKFZ), Heidelberg, Germany; 11 Institute for Occupational and Maritime Medicine (ZfAM), University Medical Center Hamburg-Eppendorf, Hamburg, Germany; 12 Department of Epidemiological Methods and Etiological Research, Leibniz Institute for Prevention Research and Epidemiology - BIPS, Bremen, Germany; 13 Department for Epidemiology, Helmholtz Centre for Infection Research (HZI), Hannover, Germany; 14 Department SHIP clinical epidemiological research, Institute for Community Medicine, University Medicine Greifswald, Greifswald, Germany; 15 Institute of Social Medicine, Epidemiology and Health Economics, Charité—Universitätsmedizin Berlin, Berlin, Germany; 16 Institute of Clinical Epidemiology and Biometry, University of Würzburg, Würzburg, Germany; 17 State Institute of Health I, Bavarian Health and Food Safety Authority, Erlangen, Germany; 18 Institute of Medical Epidemiology, Biometrics, and Informatics, Medical Faculty of the Martin-Luther-University Halle-Wittenberg, Halle (Saale), Germany; 19 Institute for Prevention and Cancer Epidemiology, Faculty of Medicine and Medical Center, University of Freiburg, Freiburg, Germany; 20 Institute of Clinical Chemistry and Laboratory Medicine, University Medicine Greifswald, Greifswald, Germany; 21 Max-Delbrueck-Center for Molecular Medicine in the Helmholtz Association (MDC), Molecular Epidemiology Research Group, Berlin, Germany; 22 Max-Delbrueck-Center for Molecular Medicine in the Helmholtz Association (MDC), Biobank Technology Platform, Berlin, Germany; 23 Charité - Universitätsmedizin Berlin, corporate member of Freie Universität Berlin and Humboldt-Universität zu Berlin, Berlin, Germany; 24 German Center for Diabetes Research (DZD), Neuherberg, Germany; 25 Charité - Universitätsmedizin Berlin, corporate member of Freie Universität Berlin and Humboldt-Universität zu Berlin, Department of Endocrinology and Metabolism, Berlin, Germany; 26 Department of Epidemiology, IUF-Leibniz Research Institute for Environmental Medicine, Düsseldorf, Germany; 27 Institute for Medical Informatics, Biometry and Epidemiology, University Hospital Essen, University Duisburg-Essen, Essen, Germany; 28 Department of Molecular Epidemiology, German Institute of Human Nutrition Potsdam-Rehbruecke, Nuthetal, Germany; 29 Institute of Nutritional Science, University of Potsdam, Nuthetal, Germany

**Keywords:** Epidemiology, Risk factors

## Abstract

**Background:**

Physical activity supports weight regulation and metabolic health, but its timing in relation to obesity and diabetes remains unclear. We aimed to assess the diurnal timing of physical activity and its association with obesity and diabetes.

**Methods:**

We cross-sectionally analyzed hip-worn accelerometry data from 61,116 participants aged 20–75 in the German National Cohort between 2015 and 2019. We divided physical activity into sex- and age-standardized quartiles of total morning (06:00–11:59), afternoon (12:00–17:59), evening (18:00–23:59), and nighttime (00:00–06:00) physical activity. Using multivariable logistic regression, we estimated associations of physical activity timing with obesity (BMI ≥ 30.0 kg/m^2^) and diabetes (self-reported or HbA1c ≥ 6.5%). We accounted for sex, age, study region, education, employment, risky alcohol use, smoking, night shift work, and sleep duration.

**Results:**

High afternoon (top vs. bottom quartile, OR: 0.36, 95% CI: 0.33–0.38) and evening physical activity (OR: 0.45, 95% CI: 0.42–0.48) showed lower obesity odds than high morning activity (OR: 0.71, 95% CI: 0.66–0.76), whereas nighttime activity increased obesity odds (OR: 1.58, 95% CI: 1.48–1.68). Associations were similar for diabetes, with the lowest odds for afternoon (OR: 0.47, 95% CI: 0.42–0.53), followed by evening (OR: 0.56, 95% CI: 0.50–0.62) and morning activity (OR: 0.80, 95% CI: 0.71–0.89), and higher odds for nighttime activity (OR: 1.43, 95% CI: 1.29–1.58). Findings were not modified by employment status, night shift work, and sleep duration.

**Conclusions:**

Our cross-sectional findings require longitudinal corroboration but suggest afternoon and evening activity provide greater metabolic health benefits than morning activity, while nighttime activity is discouraged.

## Introduction

Endogenous circadian rhythms are essential in regulating human metabolism and metabolic balance throughout the day [1]. Lipid metabolism, adipokine production, and energy homeostasis all fluctuate based on time of day [2]. Synchronization between the body’s endogenous circadian rhythms and exogenous environmental cues, such as light and dark cycles, is crucial for maintaining health [3]. However, misalignment between individuals’ daily schedules and their circadian clocks, often due to personal choices or constraints, can disrupt these rhythms, increasing the risk of metabolic and other diseases [4]. Engaging in physical activity at night, for example, may negatively affect metabolism [5], although physical activity generally helps synchronize circadian rhythms, e.g., through modulation of circadian gene expression, inducing a phase shift of core clock genes [6].

The negative effects of circadian misalignment on metabolic processes is fairly well documented. For example, animal studies have linked circadian disruption to obesity, hyperglycemia, and hypoinsulinemia [7, 8]. Similarly, evidence in humans associates circadian misalignment with negative cardiometabolic outcomes in humans, including weight gain, impaired glucose tolerance, reduced insulin sensitivity, and diabetes [9–12].

Recent research highlights that the timing of physical activity significantly impacts metabolic outcomes, with afternoon and evening activity offering greater benefits than morning activity. Specifically, studies consistently link activity later in the day to lower body mass index (BMI) [13] and improved glycemic control [14–19]. Among individuals with diabetes, physical activity in the afternoon or evening is related to lower glycated hemoglobin (HbA1c) levels and reduced cardiovascular disease risk, compared to other times of the day [20, 21]. Conversely, nighttime physical activity has been associated with increased cardiovascular and overall mortality [22, 23]. However, epidemiologic research relating nighttime activity to metabolic disorders is limited to one small study, which reported that older adults participating in nighttime activity had larger body size and higher blood glucose levels [13].

While these findings suggest a link between physical activity timing and metabolic outcomes, evidence on the impact of nighttime physical activity on obesity and diabetes remains sparse. To address this research gap, we conducted a large cross-sectional study to investigate accelerometer-measured physical activity across different times of day, including nighttime, in relation to obesity and diabetes. We aimed at identifying specific times of the day when physical activity is potentially most strongly associated with metabolic health.

## Subjects, materials and methods

### Study population

The German National Cohort (NAKO) is a population-based prospective cohort that recruited 205,415 men and women aged 20–75 years across 18 study centers in urban, industrialized, and rural regions of Germany. The study’s design has been described elsewhere [24]. Participants were randomly selected through an age- and sex-stratified sampling process, with 10,000 participants per 10-year age group from 20 to 39 years, and 26,667 per group aged 40–69 years. The baseline response proportion was 17%. Baseline assessments included touchscreen questionnaires, interviews, physical and functional measurements, and biosample collection. In an extended examination module, 70,005 participants wore a triaxial accelerometer over a period of seven days. Ethics approval was obtained from relevant local committees. All participants provided written informed consent.

We excluded participants without valid accelerometry data (<16 h of wear time per day, no valid weekend day and <2 valid weekdays), underweight individuals (BMI < 18.5 kg/m^2^), and those with missing outcome data, resulting in 61,114 participants for analysis (Supplementary Fig. 1).

### Physical activity assessment and data processing

We measured physical activity using triaxial ActiGraph models (GT3X + , wGT3X + , and wGT3X-BT; ActiGraph, Pensacola, FL, USA). The device was worn on the right hip during usual activities, starting the day following the visit for seven full days of measurement. To be precise, participants were instructed to wear the device throughout the day and perform their activities as usual. The device should only be removed when in contact with water for more than 30 min. Data processing details are described elsewhere [25]. The mean amplitude deviation (MAD), a measure of physical activity volume, was calculated from the raw data, aggregated to hourly averages, and expressed in milligravity (m*g*) units. MAD may be superior to other common metrics for hip-worn data as it is less sensitive to calibration errors [26, 27].

We divided physical activity into sex- and age-specific (5-year increments) quartiles of total activity during the day (06:00–23:59) and night (00:00–06:00). Daytime was further divided into morning (06:00–11:59), afternoon (12:00–17:59), and evening (18:00–23:59), consistent with previous methodologies [18, 20].

### Outcome measures: obesity and diabetes

We examined the association between physical activity timing and the prevalence of obesity (BMI ≥ 30.0 kg/m^2^) and diabetes (self-report of previous physician’s diagnoses or HbA1c levels ≥6.5%), following World Health Organization guidelines [28]. BMI was calculated as weight divided by height in meters squared. Body height and weight were measured using the seca Stadiometer 274 and the seca Body Composition Analyzer (mBCA) 515, respectively, to the nearest 0.1 cm and 0.1 kg (secaGmbH & Co. KG, Hamburg, Germany), with participants in their underwear, without shoes. HbA1c was analyzed by means of high-performance liquid chromatography, capillary electrophoresis, immunoturbidimetry, or immunoassay on EDTA blood samples from all NAKO study centers using the Tosoh G8 and G11 HPLC Analyzer (Tosoh Bioscience Inc., San Francisco, USA), Hemoglobin Testing Systems (D-100, VariantTM II, VariantTM II Turbo; Bio-Rad Laboratories, Hercules, USA), DxC 800 (Beckman Coulter, Brea, USA), Cobas c502 (Roche Diagnostics, Rotkreuz, Switzerland), Capillarys (Sebia, Lisses, France), and Dimension Vista 1500 (Siemens Healthineers, Erlangen, Germany). We disregarded HbA1c levels above the 99th percentile to exclude extreme outliers.

### Covariates

We examined the relations of physical activity timing to obesity and diabetes, adjusting for sex, age, and study region (south-east, south-west, north-east, north-west, west, central Germany, and the Berlin area), education (still in school; primary school, lower or intermediate secondary school; completed vocational/company training; college entrance qualification/vocational completion; university degree or doctorate), employment (employed; unemployed; outside the labor force), risky alcohol use (no; yes; according to established screening thresholds, i.e., ≥4 points for men and ≥3 points for women in the Alcohol Use Disorders Identification Test-Consumption (AUDIT-C) [29]), smoking (never; former; current), night shift work (never/occasionally; regularly/always), and sleep duration ( ≤ 7 h; 7–9 h, ≥9 h; estimated from self-reported usual wake-up time and bedtime).

### Statistical analysis

Statistical analyses included mean values, standard deviations, and Spearman correlation coefficients for physical activity across different time periods. We employed logistic regression models to estimate odds ratios (ORs) with 95% confidence intervals (CIs) for sex- and age-specific quartiles of physical activity in each time period in relation to obesity and diabetes. Model 1 was adjusted for sex, age, and study region, while model 2 further included education, employment, alcohol use, smoking, night shift work, and sleep duration. Missing covariate data were handled using the missing indicator method, where participants with missing covariates were not excluded but coded as missing to maintain statistical power.

We examined the synergy between daytime and nighttime physical activity by assessing the interaction on multiplicative and additive scales, the latter using the Relative Excess Risk due to Interaction (RERI) and the delta method [30]. To adjust for multiple comparisons, we applied Bonferroni correction (α/6). We estimated p-values using likelihood ratio tests and a 5% statistical significance level. Model fit was evaluated using R^2^ and the Akaike Information Criterion. Influential observations were analyzed using Cook’s distance, studentized residuals, and hat values [31], while the linearity assumption was checked with restricted cubic splines by testing whether the coefficients of the second and third spline transformations equaled zero [32].

To account for the compositional nature of physical activity during the day, we also examined the impact of substituting physical activity from one time period to another, following the approach of a previous study [33]. Specifically, we quantified the odds of obesity and diabetes when replacing 60 m*g* of morning activity with equivalent amounts of afternoon or evening activity. Daytime activity periods were standardized by dividing each time period-specific total physical activity by 60, equivalent to approximately one standard deviation of activity per period. Models were mutually adjusted for afternoon and evening activity, along with total daytime activity (sum of morning, afternoon, and evening). The ORs represent the effect of substituting one unit of morning activity with that from another period, rotating the reference period to obtain results for each.

We conducted additional analyses to test the robustness of our findings. First, we modeled time period-specific physical activity as a continuous measure to assess the shapes of the associations with obesity and diabetes. When the linearity assumption was violated, we used restricted cubic splines with knots at the 0.05, 0.35, 0.65, and 0.95 quantiles [32]. Second, we shifted the time periods back and forward by one hour to assess the robustness of the associations. Third, we stratified by employment status to consider the impact of work-related activity. Fourth, we analyzed participants who did not commonly work night shifts to rule out the influence of shift work on nighttime activity. Fifth, we assessed potential sex differences by stratifying the analyses by sex. Sixth, although we viewed BMI as a mediator in the association between physical activity timing and diabetes [34], we conducted an additional analysis including BMI as a covariate to assess its impact. Seventh, we used linear regression to evaluate the relations of physical activity timing to BMI and HbA1c as continuous variables. Eight, we restricted the diabetes analysis to HbA1c measurements from 12 NAKO study centers which were originally re-analyzed centrally to reassure data quality. Finally, we examined the influence of potential non-wear during the night by including the average number of measurement hours in the models as well as the midpoint of self-reported sleep to investigate the potential influence of sleep timing.

All analyses were performed using R version 4.2.3 [35]. Restricted cubic splines were modeled with the rms package [36].

## Results

The study population had an average age of 50 (±13) years, with a nearly equal gender distribution (52% women). Among the participants, 10,239 (17%) had obesity, 1904 (3%) had diabetes, and 1883 (3%) had both conditions. The average overall physical activity over 24 h (MAD) was 19.9 ( ± 6.1) m*g*.

We analyzed participant characteristics to identify potential confounding factors. Participants with higher daytime physical activity (06:00–23:59) were more likely to have high education, be employed, and have never smoked, but they also tended to exhibit risky alcohol consumption compared to those with lower daytime physical activity. In contrast, participants with greater nighttime physical activity (00:00–05:59) tended to have lower education, shorter sleep duration, engage in shift work, and be current smokers compared to those with lower nighttime activity (Table 1).

**Table 1:** Baseline characteristics (2014 to 2019) of NAKO participants (*N* = 61,116) by sex- and age-standardized quartiles of daytime and nighttime physical activity.

Characteristic	Full cohort	Daytime physical activity		Nighttime physical activity	
		1st quartile	4th quartile	1st quartile	4th quartile
	N (%) / Mean (SD)				
**N**	61,116	15,283	15,288	15,283	15,288
**Age (years)**	50.1 (12.5)	50.2 (12.5)	50.1 (12.5)	50.2 (12.5)	50.1 (12.5)
**Sex**					
Men	29,407 (48.1%)	7354 (48.1%)	7356 (48.1%)	7354 (48.1%)	7356 (48.1%)
Women	31,709 (51.9%)	7929 (51.9%)	7932 (51.9%)	7929 (51.9%)	7932 (51.9%)
**Daytime activity (m*g*)**	449.6 (140.8)	294.8 (55.6)	631.3 (109.5)	433.5 (134.3)	472.6 (147.3)
**Morning activity (m*g*)**	150.8 (67.2)	96.7 (30.8)	217.1 (79.3)	149.3 (62.4)	156.2 (75.9)
**Afternoon activity (m*g*)**	190.9 (68.7)	125.2 (32.0)	265.5 (69.9)	185.9 (67.1)	196.9 (70.1)
**Evening activity (m*g*)**	107.9 (54.2)	72.9 (28.2)	148.7 (68.5)	98.3 (49.8)	119.5 (60.3)
**Nighttime activity (m*g*)**	27.2 (21.8)	25.2 (19.6)	29.9 (24.4)	15.8 (1.4)	48.9 (34.9)
**Body mass index (kg/m**^**2**^**)**	26.5 (4.8)	28.3 (5.8)	25.1 (3.8)	26.2 (4.4)	26.7 (5.0)
**HbA1c (%)**	5.5 (0.6)	5.5 (0.7)	5.4 (0.4)	5.4 (0.5)	5.5 (0.6)
Missing	2360	667	517	593	596
**Education level**					
Still in school	1197 (2.0%)	324 (2.1%)	312 (2.0%)	277 (1.8%)	325 (2.1%)
Primary school, lower or intermediate secondary school	1227 (2.0%)	415 (2.7%)	259 (1.7%)	257 (1.7%)	409 (2.7%)
Completed vocational/company training	15,051 (24.6%)	4111 (26.9%)	3677 (24.1%)	3498 (22.9%)	4310 (28.2%)
College entrance qualification and vocational completion	7596 (12.4%)	1944 (12.7%)	1815 (11.9%)	1873 (12.3%)	2047 (13.4%)
University degree or doctorate	31,915 (52.2%)	7327 (47.9%)	8227 (53.8%)	8387 (54.9%)	7113 (46.5%)
Missing	4130 (6.8%)	1162 (7.6%)	998 (6.5%)	991 (6.5%)	1084 (7.1%)
**Employment**					
Employed	47,423 (77.6%)	10,911 (71.4%)	12,293 (80.4%)	11,624 (76.1%)	12,350 (80.8%)
Unemployed	1598 (2.6%)	732 (4.8%)	252 (1.6%)	375 (2.5%)	354 (2.3%)
Outside the labor force	11,703 (19.1%)	3516 (23.0%)	2649 (17.3%)	3190 (20.9%)	2493 (16.3%)
Missing	392 (0.6%)	124 (0.8%)	94 (0.6%)	94 (0.6%)	91 (0.6%)
**Sleep duration (hours)**					
≤7 h	13,638 (22.3%)	3215 (21.0%)	3634 (23.8%)	1837 (12.0%)	5226 (34.2%)
7–9 h	31,825 (52.1%)	7387 (48.3%)	8223 (53.8%)	9294 (60.8%)	6213 (40.6%)
≥9 h	8928 (14.6%)	2840 (18.6%)	1821 (11.9%)	2592 (17.0%)	1901 (12.4%)
Missing	6725 (11.0%)	1841 (12.0%)	1610 (10.5%)	1560 (10.2%)	1948 (12.7%)
**Night shift work**					
Never / unregularly	35,506 (58.1%)	8098 (53.0%)	9179 (60.0%)	9,226 (60.4%)	8198 (53.6%)
Regularly/ always	2387 (3.9%)	631 (4.1%)	608 (4.0%)	201 (1.3%)	1452 (9.5%)
Missing	23,223 (38.0%)	6554 (42.9%)	5501 (36.0%)	5856 (38.3%)	5638 (36.9%)
**Risky alcohol drinking**					
No	38,839 (63.5%)	10,184 (66.6%)	9414 (61.6%)	10,498 (68.7%)	8963 (58.6%)
Yes	20,724 (33.9%)	4669 (30.6%)	5512 (36.1%)	4411 (28.9%)	5879 (38.5%)
Missing	1553 (2.5%)	430 (2.8%)	362 (2.4%)	374 (2.4%)	446 (2.9%)
**Smoking status**					
Never	28,579 (46.8%)	6440 (42.1%)	7607 (49.8%)	7958 (52.1%)	6268 (41.0%)
Former	19,986 (32.7%)	4736 (31.0%)	5219 (34.1%)	4993 (32.7%)	4908 (32.1%)
Current	11,106 (18.2%)	3717 (24.3%)	2128 (13.9%)	1982 (13.0%)	3697 (24.2%)
Missing	1445 (2.4%)	390 (2.6%)	334 (2.2%)	350 (2.3%)	415 (2.7%)

*HbA1c* glycated hemoglobin.

Daytime physical activity correlated most strongly with afternoon activity (r = 0.85), followed by morning (r = 0.72) and evening activities (r = 0.63). Morning activity correlated only moderately or weakly with afternoon (r = 0.48) and evening activity (r = 0.16). Nighttime activity had minimal correlation with daytime activity, with the highest being between evening and nighttime activity (r = 0.22), negligible correlation with morning activity (r = 0.03), and a low correlation with afternoon activity (r = 0.10) (Supplementary Table 1).

### Timing of physical activity and obesity

Higher levels of daytime physical activity were consistently associated with lower odds of obesity compared to lower levels of daytime activity. After adjusting for sex, age, and study region, the OR for obesity when comparing individuals in the highest versus the lowest quartile of daytime physical activity was 0.22 (95% CI: 0.21–0.24). Further adjustments for education, employment status, alcohol use, smoking status, night shift work frequency, and sleep duration did not alter the association (Table 2). In contrast, higher compared to lower nighttime physical activity was associated with increased odds of obesity (OR in highest versus lowest quartile of nighttime activity: 1.61, 95% CI: 1.52–1.71). This relation was slightly attenuated after adjusting for covariates, but it remained statistically significant (OR: 1.51, 95% CI: 1.42–1.61). We observed an additive interaction between daytime and nighttime activity (*p* value for interaction: 0.0004) but no multiplicative interaction (Table 2). High daytime activity attenuated but did not fully eliminate the increased odds related to high nighttime activity (Supplementary Table 2). When modeling physical activity on a continuous scale, we noted non-linear relationships between both daytime and nighttime physical activity and obesity (Supplementary Fig. 2).

**Table 2:** Odds ratios and 95% confidence intervals (CI) for timing of physical activity in relation to obesity and diabetes (*N* = 61,116).

Obesity					
		Model 1		Model 2	
Physical activity timing	Cases	Odds ratio	95% CI	Odds ratio	95% CI
**Daytime**					
1st Quartile	4980	1.00	–	1.00	–
2nd Quartile	3229	0.54	0.51, 0.57	0.54	0.51, 0.57
3rd Quartile	2321	0.35	0.33, 0.37	0.35	0.33, 0.37
4th Quartile	1592	0.22	0.21, 0.24	0.22	0.21, 0.24
*P for trend*		<0.0001		<0.0001	
**Nighttime**					
1st Quartile	2620	1.00	–	1.00	–
2nd Quartile	2944	1.23	1.16, 1.31	1.22	1.15, 1.30
3rd Quartile	3264	1.44	1.36, 1.53	1.41	1.33, 1.50
4th Quartile	3294	1.61	1.52, 1.71	1.51	1.42, 1.61
*P for trend*		<0.0001		<0.0001	
**P for Interaction**					
Multiplicative scale		0.0203		0.0752	
Additive scale		<0.0001		0.0004	
**Daytime**					
1st Quartile	1572	1.00	–	1.00	–
2nd Quartile	928	0.55	0.50, 0.60	0.59	0.54, 0.64
3rd Quartile	731	0.42	0.38, 0.46	0.46	0.41, 0.50
4th Quartile	556	0.31	0.28, 0.34	0.34	0.30, 0.37
*P for trend*		<0.0001		<0.0001	
**Nighttime**					
1st Quartile	847	1.00	–	1.00	–
2nd Quartile	951	1.18	1.07, 1.30	1.18	1.07, 1.30
3rd Quartile	966	1.22	1.10, 1.34	1.22	1.10, 1.34
4th Quartile	1023	1.39	1.26, 1.53	1.37	1.24, 1.51
*P for trend*		<0.0001		<0.0001	
**P for interaction**					
Multiplicative scale		0.0233		0.0215	
Additive scale		0.0001		0.0003	

Model 1: Adjusted for sex, age, and study region.

Model 2: Model 1 and adjusted for education, employment status, risky alcohol use, smoking status, night shift work frequency, and sleep duration.

Note: Quartiles are time period-, sex- and age-specific.

Median values for daytime physical activity:

Q1: 301 m*g*; Q2: 398 m*g*; Q3: 479 m*g*; Q4: 607 m*g*.

Median values for nighttime physical activity:

Q1: 16 m*g*; Q2: 19 m*g*; Q3: 24 m*g*; Q4: 38 m*g*.

**Fig. 1:**
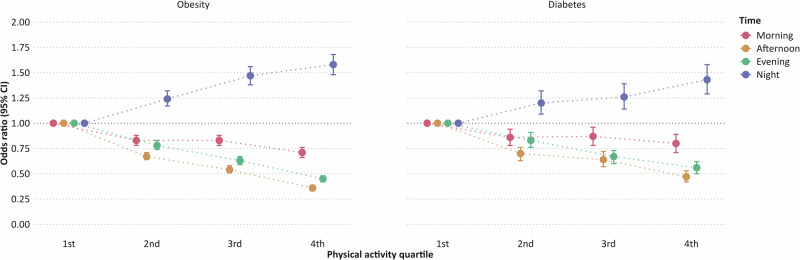
Odds ratios and 95% confidence intervals (CI) for timing of physical activity in relation to obesity (left) and diabetes (right). Adjusted for sex, age, study region, education, employment status, alcohol use, smoking status, night shift work frequency, and sleep duration.

All periods of daytime physical activity were inversely associated with obesity in a linear fashion, regardless of model adjustments (Table 3, Fig. 1). Afternoon physical activity showed the strongest inverse relation when comparing the highest versus the lowest quartiles (OR: 0.36, 95% CI: 0.33–0.38), followed by evening (OR: 0.45, 95% CI: 0.42–0.48) and morning activity (OR: 0.71, 95% CI: 0.66–0.76). Statistically significant multiplicative interactions were found between daytime activity periods (Supplementary Tables 3–5). Specifically, afternoon and evening activity weakened but did not eliminate the odds related to morning activity, and afternoon activity similarly reduced but did not nullify the odds associated with evening activity. Morning activity was linearly associated with lower obesity, while afternoon and evening activity showed non-linear inverse associations, with steeper slopes compared to morning activity (Supplementary Fig. 3).

**Table 3:** Odds ratios and 95% confidence intervals (CI) for timing of physical activity in relation to obesity (*N* = 61,116).

Physical activity timing	Cases	Model 1		Model 2	
		Odds ratio	95% CI	Odds ratio	95% CI
**Morning**					
1st Quartile	4091	1.00	–	1.00	–
2nd Quartile	3090	0.83	0.79, 0.88	0.83	0.78, 0.88
3rd Quartile	2757	0.84	0.79, 0.89	0.83	0.78, 0.88
4th Quartile	2184	0.74	0.69, 0.79	0.71	0.66, 0.76
*P for trend*		<0.0001		<0.0001	
**Afternoon**					
1st Quartile	4823	1.00	–	1.00	–
2nd Quartile	3187	0.67	0.64, 0.71	0.67	0.64, 0.71
3rd Quartile	2506	0.55	0.52, 0.59	0.54	0.51, 0.58
4th Quartile	1606	0.36	0.34, 0.39	0.36	0.33, 0.38
*P for trend*		<0.0001		<0.0001	
**Evening**					
1st Quartile	4498	1.00	–	1.00	–
2nd Quartile	3314	0.75	0.71, 0.80	0.78	0.74, 0.83
3rd Quartile	2565	0.60	0.56, 0.63	0.63	0.59, 0.67
4th Quartile	1745	0.42	0.39, 0.45	0.45	0.42, 0.48
*P for trend*		<0.0001		<0.0001	
**Nighttime**					
1st Quartile	2620	1.00	–	1.00	–
2nd Quartile	2944	1.25	1.18, 1.33	1.24	1.17, 1.32
3rd Quartile	3264	1.50	1.42, 1.60	1.47	1.38, 1.56
4th Quartile	3294	1.69	1.59, 1.80	1.58	1.48, 1.68
*P for trend*		<0.0001		<0.0001	

Model 1: Adjusted for sex, age, and study region.

Model 2: Model 1 and adjusted for education, employment status, alcohol use, smoking status, night shift work frequency, and sleep duration.

Note: Quartiles are time period-, sex-, and age-specific.

Median values for morning physical activity:

Q1: 86 m*g*; Q2: 123 m*g*; Q3: 158 m*g*; Q4: 221 m*g*.

Median values for afternoon physical activity:

Q1: 120 m*g*; Q2: 164 m*g*; Q3: 202 m*g*; Q4: 265 m*g*.

Median values for evening physical activity:

Q1: 57 m*g*; Q2: 86 m*g*; Q3: 113 m*g*; Q4: 163 m*g*.

Median values for night physical activity:

Q1: 16 m*g*; Q2: 19 m*g*; Q3: 24 m*g*; Q4: 38 m*g*.

### Timing of physical activity and diabetes

Higher levels of daytime physical activity compared to lower levels were associated with lower odds of diabetes (OR: 0.31, 95% CI: 0.28–0.34). This relation remained virtually unaltered after adjusting for education, employment status, alcohol use, smoking status, night shift work frequency, and sleep duration (Table 2). By comparison, higher compared to lower nighttime physical activity was associated with higher odds of diabetes (OR: 1.39, 95% CI: 1.26–1.53), and further adjustments had little impact (OR: 1.37, 95% CI: 1.24–1.51). We noted both multiplicative (*p* value: 0.0215) and additive (*p* value: 0.0003) interactions between daytime and nighttime activity (Table 2), with high daytime activity nullifying the odds associated with high nighttime activity (Supplementary Table 1). On a continuous scale, both daytime and nighttime physical activity showed non-linear associations with diabetes, which were largely consistent with our findings for obesity (Supplementary Fig. 2).

Higher daytime physical activity was consistently associated with lower odds of diabetes across all periods and model adjustments, although the relations were slightly less pronounced than those for obesity (Table 4, Fig. 1). We observed significant multiplicative interactions between different periods of daytime activity (Supplementary Tables 3, 6, 7). Afternoon and evening activity both nullified the odds related to morning activity, and afternoon activity similarly eliminated the odds related to evening activity (i.e., CIs included the null value). When modeled continuously, time period-specific physical activity showed non-linear associations with diabetes, with steeper slopes for afternoon and evening activity compared to morning activity (Supplementary Fig. 3).

**Table 4:** Odds ratios and 95% confidence intervals (CI) for timing of physical activity in relation to diabetes (*N* = 61,116).

Physical activity timing	Cases	Model 1		Model 2	
		Odds ratio	95% CI	Odds ratio	95% CI
**Morning**					
1st Quartile	1290	1.00	–	1.00	–
2nd Quartile	937	0.83	0.76, 0.91	0.86	0.78, 0.94
3rd Quartile	845	0.85	0.77, 0.93	0.87	0.78, 0.96
4th Quartile	715	0.79	0.71, 0.88	0.80	0.71, 0.89
*P for trend*		<0.0001		0.0001	
**Afternoon**					
1st Quartile	1529	1.00	–	1.00	–
2nd Quartile	925	0.68	0.62, 0.74	0.70	0.63, 0.76
3rd Quartile	785	0.62	0.56, 0.69	0.64	0.57, 0.70
4th Quartile	548	0.46	0.41, 0.52	0.47	0.42, 0.53
*P for trend*		<0.0001		<0.0001	
**Evening**					
1st Quartile	1431	1.00	–	1.00	–
2nd Quartile	1031	0.79	0.72, 0.86	0.83	0.76, 0.91
3rd Quartile	752	0.61	0.55, 0.67	0.67	0.60, 0.73
4th Quartile	573	0.50	0.45, 0.56	0.56	0.50, 0.62
*P for trend*		<0.0001		<0.0001	
**Nighttime**					
1st Quartile	847	1.00	–	1.00	–
2nd Quartile	951	1.20	1.08, 1.32	1.20	1.09, 1.32
3rd Quartile	966	1.26	1.15, 1.39	1.26	1.14, 1.39
4th Quartile	1023	1.46	1.32, 1.61	1.43	1.29, 1.58
*P for trend*		<0.0001		<0.0001	

Model 1: Adjusted for sex, age, and study region.

Model 2: Model 1 and adjusted for education, employment status, alcohol use, smoking status, night shift work frequency, and sleep duration.

Note: Quartiles are time period-, sex-, and age-specific.

Median values for morning physical activity:

Q1: 86 mg; Q2: 123 mg; Q3: 158 mg; Q4: 221 mg.

Median values for afternoon physical activity:

Q1: 120 mg; Q2: 164 mg; Q3: 202 mg; Q4: 265 mg.

Median values for evening physical activity:

Q1: 57 mg; Q2: 86 mg; Q3: 113 mg; Q4: 163 mg.

Median values for night physical activity:

Q1: 16 mg; Q2: 19 mg; Q3: 24 mg; Q4: 38 mg.

### Substitution models

Reallocating one unit of physical activity (60 m*g*) from the morning to the continuous activity spectrum of the afternoon or the evening was associated with lower odds of obesity (OR: 0.77, 95% CI: 0.74–0.80). Conversely, shifting afternoon or evening activity to morning was related to increased odds of obesity (afternoon to morning: OR: 1.47, 95% CI: 1.31 to 1.65; evening to morning: OR: 1.46, 95% CI: 1.30–1.63). No association was observed when reallocating afternoon to evening activity. The substitution model results for diabetes mirrored those for obesity (Supplementary Table 8). Allocating physical activity to the morning showed slight, non-linear positive associations with both obesity and diabetes (Supplementary Fig. 4).

### Sensitivity analyses

Shifting the time periods back by one hour slightly attenuated the odds related to morning, afternoon and nighttime activity and increased the odds related to evening activity for both obesity and diabetes. Shifting the time periods forward by one hour slightly increased the odds related to morning activity and attenuated the odds related to afternoon, evening, and nighttime activity for both obesity and diabetes (Supplementary Table 9). Stratifying by employment status revealed no meaningful differences in the associations between physical activity timing and obesity. This was true for both daytime activity (e.g., afternoon activity, employed: OR: 0.39, 95% CI: 0.36–0.42; unemployed: OR: 0.34, 95% CI: 0.22–0.51) and nighttime physical activity (employed: OR: 1.50, 95% CI: 1.39–1.62; unemployed: OR: 1.72, 95% CI: 1.18–2.51; Supplementary Table 10). Results for diabetes were similarly consistent when stratified by employment status (Supplementary Table 11). Excluding night shift workers yielded similar findings (Supplementary Table 12). Stratifying the analyses by sex revealed that the associations with obesity were slightly more pronounced in women than in men (Supplementary Table 13). Including BMI in the model attenuated the odds of diabetes but had little overall impact, except for morning activity, which lost statistical significance (Supplementary Table 14). When analyzing BMI and HbA1c as continuous variables, the relation of physical activity timing to BMI paralleled the main results (Supplementary Table 15). Restricting the analysis to centrally analyzed HbA1c measurements yielded consistent results (Supplementary Table 16). Lastly, neither additional adjustment for the number of measurement hours nor for the sleep midpoint modified the main results (Supplementary Tables 17, 18).

## Discussion

The primary findings of our study indicate that physical activity in the afternoon and evening is more strongly inversely associated with both obesity and diabetes compared to morning activity. In contrast, nighttime physical activity is positively related to obesity and diabetes. These associations persisted across different levels of employment status, shift work, and sleep duration. In further analyses using substitution models, replacing afternoon or evening activity with morning activity was associated with higher odds of obesity and diabetes. Since overall physical activity levels were held constant (mutual adjustment for overall levels), any increase in morning activity corresponded to a decrease in afternoon or evening activity. This suggests that although morning activity is not harmful, afternoon and evening activity provide significantly greater health benefits. This was also indicated by the non-linear continuous relationships between physical activity timing and obesity and diabetes, with steeper slopes observed for afternoon and evening activity compared to morning activity. Additional interaction analyses revealed that afternoon activity partially diminished the beneficial effects of morning activity, indicating a potential ceiling effect, where increasing both morning and afternoon activity beyond certain levels may not provide additional advantages. Further analyses also suggested that higher levels of daytime activity could offset the adverse effects of nighttime activity on diabetes but not entirely on obesity.

The biological mechanisms linking physical activity timing to metabolic health are not yet fully understood. However, exercise capacity and the associated metabolic pathways follow a diurnal rhythm, and timing plays a crucial role in enhancing the beneficial metabolic effects of exercise. Late-day exercise, in particular, is associated with peak mitochondrial function in skeletal muscle oxidative metabolism, resulting in significant metabolic benefits [6, 37]. Moreover, afternoon and evening physical activity improves circulating levels of blood glucose, insulin, and triglycerides more effectively than activity performed earlier in the day [14–19]. In both healthy and individuals with pre-diabetes, plasma glucose clearance is more efficient in the morning compared to the evening following similar glucose intake, as insulin sensitivity decreases later in the day [38, 39]. Consequently, exercising in the late afternoon or evening may be particularly beneficial for improving glycemic control when insulin sensitivity is at its lowest. Exercise at this time helps counteract reduced insulin sensitivity by increasing glucose uptake in muscle through insulin-independent pathways, such as AMPK activation and contraction-induced GLUT4 translocation [40].

The association between nighttime physical activity and increased odds of obesity and diabetes may stem from exercise’s role as *zeitgeber* – an external cue that, when misaligned with endogenous rhythms, can disrupt metabolic regulation [3]. For example, a small study in elderly individuals reported that higher activity between midnight and 5:00 AM was associated with a 3% increase in BMI and a 2% increase in HbA1c levels [13]. Additionally, research indicates that individuals with a late chronotype (“night owls”) tend to have higher blood glucose levels, an increased risk of diabetes, and poorer glycemic control compared to those with an early chronotype (“larks”) [41, 42]. Experimental studies have shown that circadian misalignment decreases leptin, increases glucose despite higher insulin levels, and inverts the cortisol rhythm, promoting insulin resistance and metabolic dysfunction [12, 43]. Exposure to light at night may further exacerbate circadian disruption [44]. However, exercise that is aligned with the circadian clock can help restore biologic rhythms and improve metabolic health [6].

The association between increased daytime physical activity and lower odds of both obesity and diabetes, along with the slight attenuation of the relation between physical activity timing and diabetes after adjusting for BMI, suggests that the effects of physical activity timing on these conditions likely operate through shared biological mechanisms. Reduced adiposity likely plays a major role in the causal pathway linking higher physical activity to a lower risk of diabetes. A meta-analysis supports this notion, showing that the inverse association between physical activity and diabetes is partly mediated by lower adiposity [45].

We explored the potential for reverse causation in our results on nighttime activity, as low-level nocturnal activity detected by accelerometers could indicate sleep disturbances – a common issue in individuals with obesity and diabetes [46, 47]. However, we found higher odds of obesity and diabetes not only in those with slight increases in nighttime activity—possibly due to sleep disturbances—but also in those in the highest quartile of nighttime activity. The latter is less likely to be related to sleep issues, as such increased activity levels require sustained movements such as walking or other purposeful activities rather than the sporadic or light movements typically associated with sleep disturbances. However, it is not clear what the underlying activities are that lead to those highest levels of nighttime activity. The magnitude of the top quartile of nighttime activity tends to indicate light activities that may exceed restless or disturbed sleep but does not necessarily include moderate or intense activities. On the other hand, the usage of aggregated hourly averages of physical activity may have covered short, intense activity bouts that ultimately result in only slightly increased overall nighttime activity. In future studies, it will be crucial to identify the causes of increased nighttime activity, and if these are due to unintentional causes, appropriate coping strategies should be encouraged.

To ensure that our results were not influenced by confounding factors known to have an adverse metabolic impact, we controlled for unemployment, night shift work, and sleep duration and sleep midpoint, all of which contribute to obesity and diabetes [9, 48]. These adjustments, as well as stratification by these factors, did not alter our findings, confirming the robustness of our findings. Additionally, we found that our results were stable when shifting the time periods by one hour. We also conducted a sub-analysis of physical activity timing in relation to continuous measures of BMI and HbA1c to fully utilize the range of data. Our findings for BMI, expressed as a continuous variable, were consistent with those for BMI categorized as obesity, suggesting that the benefits of daytime activity and the negative effects of nighttime activity apply across the BMI spectrum, including individuals with overweight or normal weight. However, the relation of physical activity timing to HbA1c, expressed as a continuous variable, was more pronounced in the afternoon and nighttime, indicating these periods may be optimal for improving glucose homeostasis. This is an important consideration for future studies on physical activity timing in relation to pre-diabetes.

Notable strengths of our study are the large cohort of men and women and the use of objective methods to assess obesity and diabetes based on standardized protocols. The use of accelerometry data allowed us to avoid recall bias and social desirability bias common in self-report measures. Additionally, the high-resolution, raw accelerometry data enabled us to perform detailed 24-h activity analyses, as opposed to relying on manufacturer-derived summary metrics. We also accounted for key potential confounding variables, particularly employment status, night shift work, and sleep duration, ruling out that our findings were driven by circadian misalignment due to these factors.

Limitations of our study include potential selection bias, as the cohort may not fully represent the general population in Germany. However, the relatively healthy and active nature of our study population likely weakens rather than overstates the observed associations. Furthermore, due to the observational nature of our study, we cannot establish causality between physical activity timing and obesity or diabetes. The cross-sectional design also limits our ability to determine temporal relationships between physical activity and these health outcomes. Interpretation of our results is restricted by the fact that we could not distinguish between occupational and leisure-time physical activity. The absolute amount of physical activity was not similarly distributed across time periods, with higher amounts of activity observed in the afternoon than during any other time period. Nevertheless, we addressed this discrepancy by assessing substitutions of fixed amounts of activity as well as examining the slopes of the associations. We were unable to account for diet or energy intake, which could have offered valuable insights into the potential modulation of the relationship between diurnal timing of physical activity and metabolic health. Lastly, reactivity bias may have influenced participants’ activity behavior, as they were conscious of wearing an accelerometer [49].

## Conclusions

In conclusion, our findings suggest that increasing daytime physical activity, especially in the afternoon and evening, is associated with less obesity and diabetes. Therefore, these time periods potentially offer the most substantial benefits for preventing obesity and diabetes. Conversely, nighttime physical activity is associated with poorer metabolic outcomes. Future longitudinal and experimental studies will be needed to confirm these findings and explore the underlying mechanisms. Encouraging physical activity remains essential for overall health, but optimizing its timing may provide a valuable, complementary strategy to prevent metabolic disease, particularly in cases where increasing exercise volume or intensity is not feasible.

## Supplementary information

Supplemental material

## Data Availability

Registered researchers from all European Union (EU) countries can apply to use the NAKO dataset by registering and applying at http://transfer.nako.de/.
