# Supplementary material for: Diurnal timing of physical activity in relation to obesity and diabetes in the German National Cohort (NAKO)
Source: Int J Obes (Lond). 2025 Jan 24;49(5):921–30. doi: 10.1038/s41366-025-01721-9 (PMC12095084; doi:10.1038/s41366-025-01721-9)
Supplement: Supplementary file 1 — Supplemental material [file 41366_2025_1721_MOESM1_ESM.docx]

Diurnal Timing of Physical Activity in Relation to Obesity and Diabetes in the German National Cohort (NAKO)

Supplementary material

[Supplementary Figure 1. Flowchart of participant inclusion and exclusion](#_Toc183769535)

[Supplementary Figure 2. Continuous daytime and nighttime physical activity and odds of obesity and diabetes](#_Toc183769536)

[Supplementary Figure 3. Continuous time period-specific physical activity and odds of obesity and diabetes](#_Toc183769537)

[Supplementary Figure 4. (Non-)linear associations in substituting physical activity between different time periods](#_Toc183769538)

[Supplementary Table 1. Pearson correlation coefficients for timing of physical activity and overall acceleration](#_Toc183769539)

[Supplementary Table 2. Combination of nighttime and daytime activity in relation to obesity and diabetes](#_Toc183769540)

[Supplementary Table 3. Multiplicative interaction between physical activity timing and obesity and diabetes](#_Toc183769541)

[Supplementary Table 4. Odds ratios and 95% confidence intervals for morning and evening activity in relation to obesity, stratified by afternoon activity](#_Toc183769542)

[Supplementary Table 5. Odds ratios and 95% confidence intervals for morning activity in relation to obesity, stratified by evening activity](#_Toc183769543)

[Supplementary Table 6. Odds ratios and 95% confidence intervals for morning activity in relation to diabetes, stratified by evening activity](#_Toc183769544)

[Supplementary Table 7. Odds ratios and 95% confidence intervals for morning and evening activity in relation to diabetes, stratified by afternoon activity](#_Toc183769545)

[Supplementary Table 8. Re-allocating physical activity from one daytime period to another](#_Toc183769546)

[Supplementary Table 9. Timing of physical activity in relation to obesity and diabetes with shifts in time period definitions](#_Toc183769547)

[Supplementary Table 10. Timing of physical activity in relation to obesity, stratified by employment status](#_Toc183769548)

[Supplementary Table 11. Timing of physical activity in relation to diabetes, stratified by employment status](#_Toc183769549)

[Supplementary Table 12. Timing of physical activity in relation to obesity and diabetes among non-night shift workers](#_Toc183769550)

[Supplementary Table 13. Timing of physical activity in relation to obesity and diabetes stratified by sex](#_Toc183769551)

[Supplementary Table 14. Timing of physical activity in relation to diabetes with additional adjustment for body mass index](#_Toc183769552)

[Supplementary Table 15. Linear regression models for physical activity timing in relation to body mass index and HbA1c levels](#_Toc183769553)

[Supplementary Table 16. Timing of physical activity in relation to diabetes (self-reported and based on HbA1c levels) when restricting the analyses to centrally analyzed HbA1c measurements](#_Toc183769554)

[Supplementary Table 17. Timing of physical activity in relation to obesity and diabetes with additional adjustment for average measurement hours](#_Toc183769555)

[Supplementary Table 18. Timing of physical activity in relation to obesity and diabetes with additional adjustment for midpoint of sleep](#_Toc183769556)

**Corresponding author:** Michael J. Stein, Tel.: +49 941 944 5216, Email: michael.stein@ukr.de, Department of Epidemiology and Preventive Medicine, University of Regensburg, Regensburg, Germany

## Flowchart of participant inclusion and exclusion

## Continuous daytime and nighttime physical activity and odds of obesity and diabetes


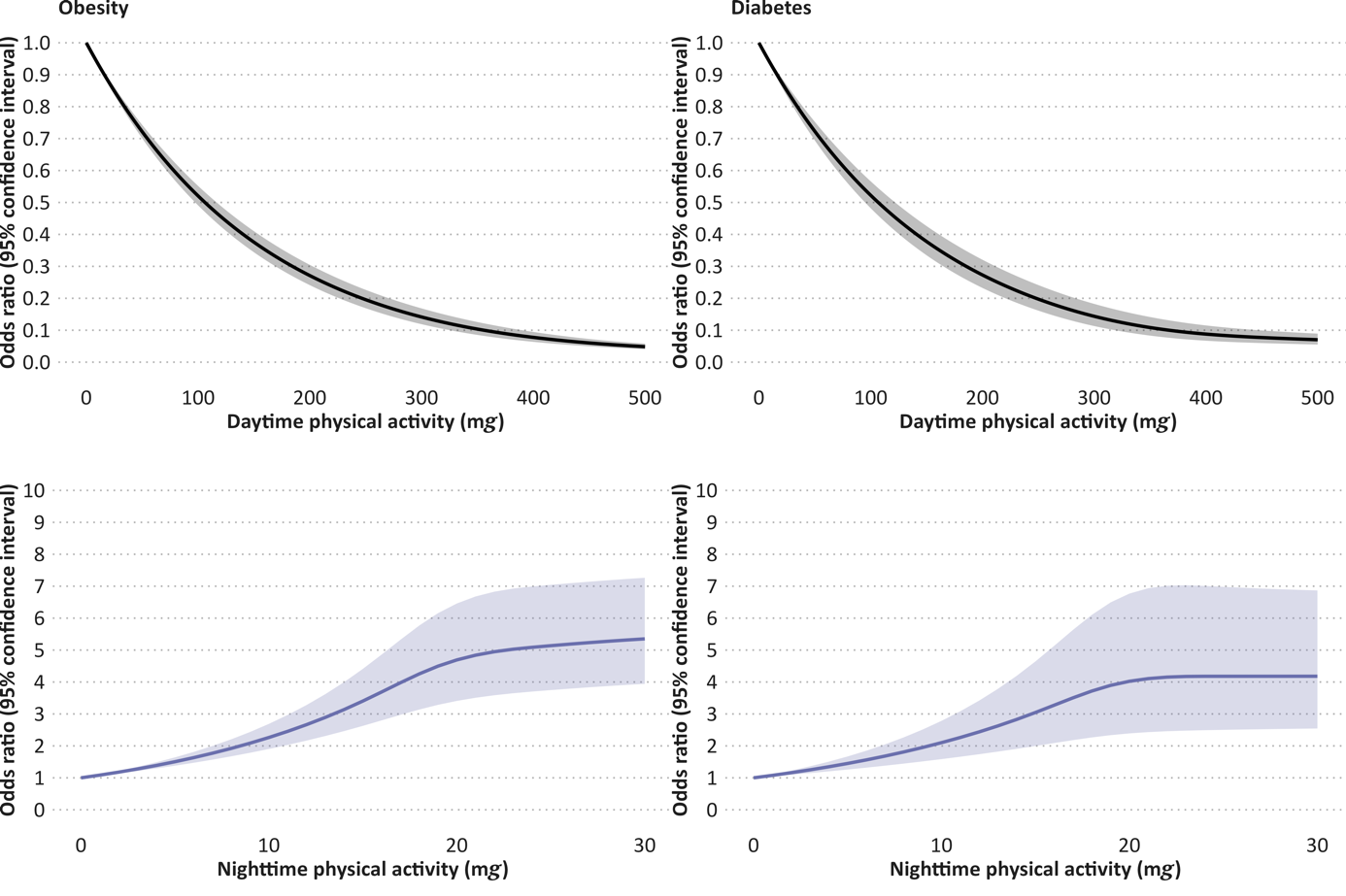


##
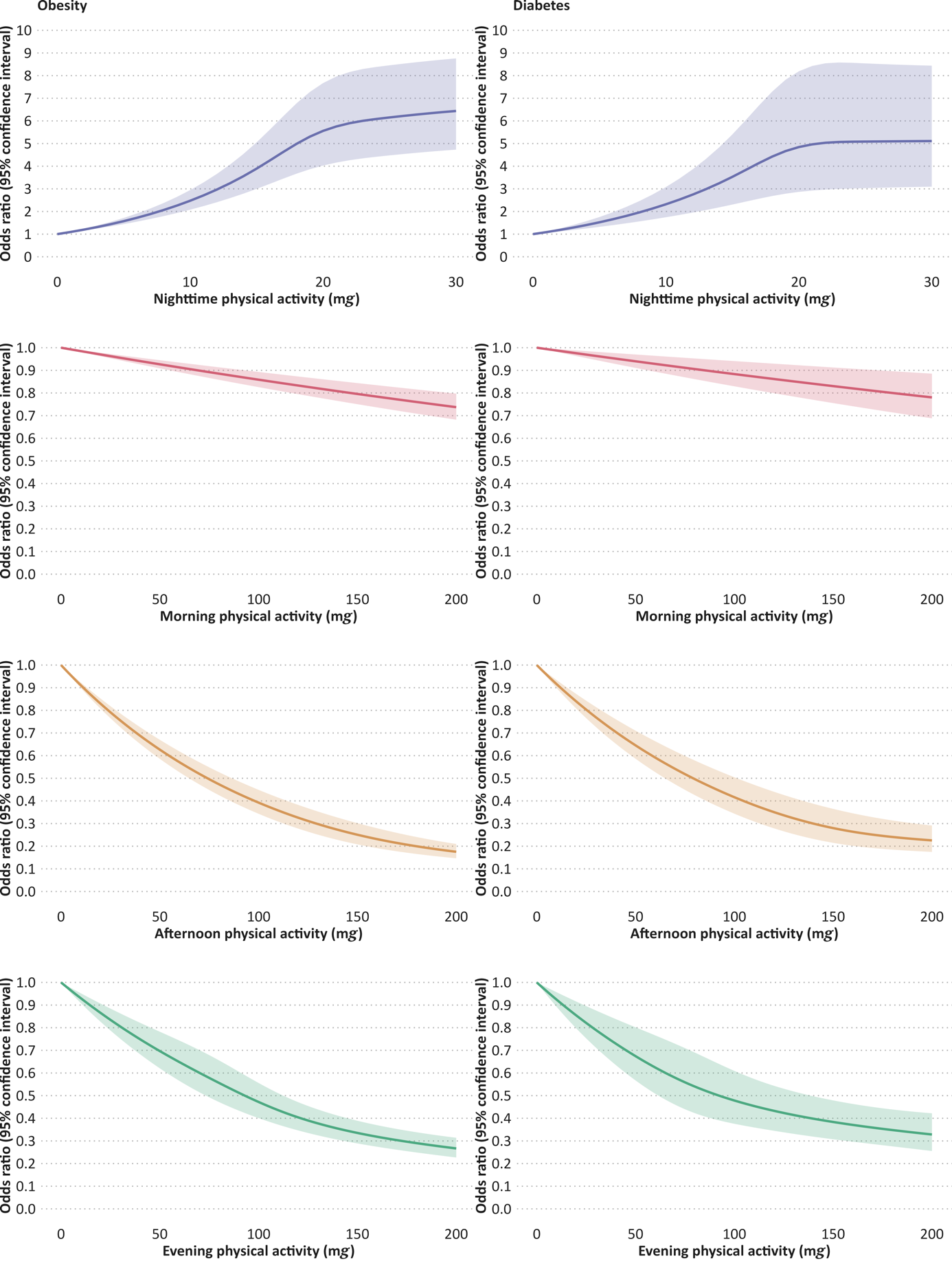
Continuous time period-specific physical activity and odds of obesity and diabetes

##
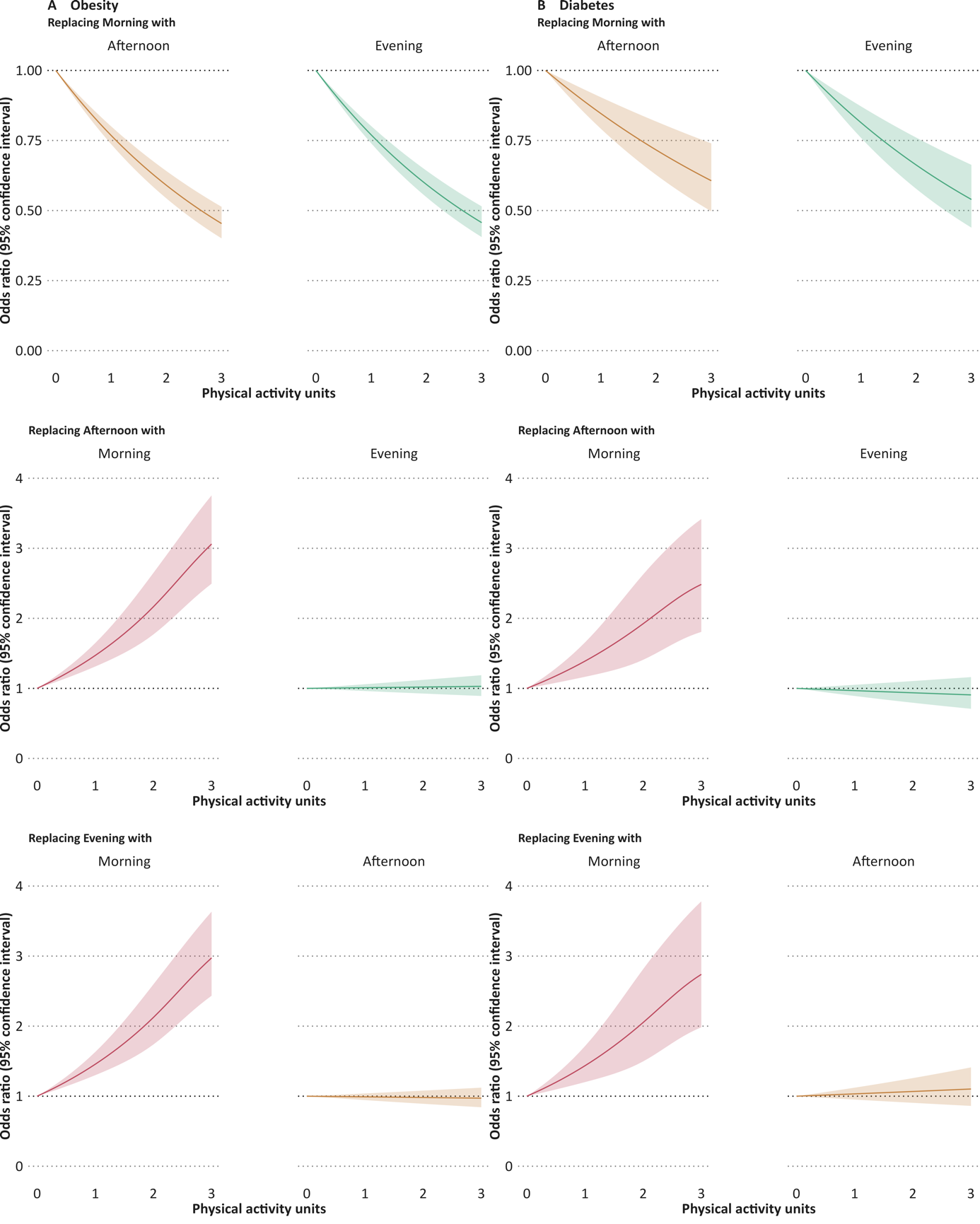
(Non-)linear associations in substituting physical activity between different time periods

# Pearson correlation coefficients for timing of physical activity and overall acceleration

|  | **Morning** | **Afternoon** | **Evening** | **Nighttime** | **Overall MAD** |
| --- | --- | --- | --- | --- | --- |
| **Overall daytime** | 0.72 | 0.85 | 0.63 | 0.15 | 0.99 |
|  | **Morning** | 0.48 | 0.16 | 0.03 | 0.71 |
|  |  | **Afternoon** | 0.41 | 0.10 | 0.83 |
|  |  |  | **Evening** | 0.22 | 0.63 |
|  |  |  |  | **Nighttime** | 0.23 |
| MAD: Mean Deviation Amplitude | | | | | |

# Combination of nighttime and daytime activity in relation to obesity and diabetes

| **Obesity** | | | |
| --- | --- | --- | --- |
| **Nighttime** | **Daytime** | **Odds ratio** | **95% Confidence interval** |
| Low | High | 1.00 | - |
| Low | Low | 2.74 | 2.56, 2.92 |
| High | High | 1.36 | 1.27, 1.46 |
| High | Low | 3.40 | 3.18, 3.63 |
| Multiplicative scale | | 0.91 | 0.84, 1.00 |
| RERI | | 0.30 | 0.12, 0.48 |
| **Diabetes** | | | |
| **Nighttime** | **Daytime** | **Odds ratio** | **95% Confidence interval** |
| Low | High | 1.00 | - |
| Low | Low | 1.82 | 1.64, 2.02 |
| High | High | 1.06 | 0.94, 1.19 |
| High | Low | 2.23 | 2.01, 2.47 |
| Multiplicative scale | | 1.16 | 1.00, 1.33 |
| RERI | | 0.35 | 0.15, 0.55 |
| RERI: Relative excess risk due to interaction  Low physical activity = activity below the median; high physical activity = activity above the median.  Models adjusted for time period, sex, age, study region, education, employment status, risky alcohol use, smoking, night shift status, and sleep duration. | | | |

# Multiplicative interaction between physical activity timing and obesity and diabetes

|  | **Obesity** | **Diabetes** |
| --- | --- | --- |
| **Interaction** | **p-value for interaction** | **p-value for interaction** |
| Nighttime * Morning | 1.0000 | 1.0000 |
| Nighttime * Afternoon | 0.4565 | 0.3101 |
| Nighttime * Evening | 1.0000 | 1.0000 |
| Morning * Afternoon | **0.0000*** | **0.0001*** |
| Morning * Evening | **0.0021*** | **0.0020*** |
| Afternoon * Evening | **0.0003*** | **0.0010*** |
| Models adjusted for time period, sex, age, study region, education, employment status, risky alcohol status, smoking, night shift status, and sleep duration.  Note: P-values were Bonferroni corrected.  * Significant interaction effects are quantified in Supplementary Tables 3-5 | | |

# Odds ratios and 95% confidence intervals for morning and evening activity in relation to obesity, stratified by afternoon activity

| **Physical activity timing** | **Afternoon activity Q1**  **N = 15,283**  **Cases = 4,823** | | **Afternoon activity Q2**  **N = 15,271**  **Cases = 3,187** | | **Afternoon activity Q3**  **N = 15,274**  **Cases = 2,506** | | **Afternoon activity Q4**  **N = 15,288**  **Cases = 1,606** | |
| --- | --- | --- | --- | --- | --- | --- | --- | --- |
| **Morning activity** | **OR** | **95% CI** | **OR** | **95% CI** | **OR** | **95% CI** | **OR** | **95% CI** |
| 1^st^ Quantile | 1.00 | - | 1.00 | - | 1.00 | - | 1.00 | - |
| 2^nd^ Quantile | 0.81 | 0.75, 0.88 | 0.91 | 0.82, 1.01 | 0.85 | 0.74, 0.98 | 0.78 | 0.63, 0.97 |
| 3^rd^ Quantile | 0.80 | 0.72, 0.90 | 0.86 | 0.77, 0.97 | 0.90 | 0.78, 1.03 | 0.89 | 0.74, 1.08 |
| 4^th^ Quantile | 0.41 | 0.35, 0.48 | 0.69 | 0.60, 0.79 | 0.84 | 0.73, 0.97 | 0.84 | 0.71, 1.01 |
| **Evening activity** |  |  |  |  |  |  |  |  |
| 1^st^ Quantile | 1.00 | - | 1.00 | - | 1.00 | - | 1.00 | - |
| 2^nd^ Quantile | 0.73 | 0.67, 0.80 | 0.82 | 0.74, 0.91 | 0.93 | 0.82, 1.06 | 0.75 | 0.63, 0.90 |
| 3^rd^ Quantile | 0.54 | 0.49, 0.61 | 0.62 | 0.55, 0.69 | 0.80 | 0.70, 0.91 | 0.72 | 0.61, 0.85 |
| 4^th^ Quantile | 0.36 | 0.31, 0.41 | 0.45 | 0.39, 0.51 | 0.56 | 0.49, 0.64 | 0.52 | 0.44, 0.61 |
| OR: Odds ratio, CI: Confidence interval  Models adjusted for time period, sex, age, and study region, education, employment status, risky alcohol status, smoking, night shift status, and sleep duration. | | | | | | | | |

# Odds ratios and 95% confidence intervals for morning activity in relation to obesity, stratified by evening activity

| **Physical activity timing** | **Evening activity Q1**  **N = 15,283**  **Cases = 4,498** | | **Evening activity Q2**  **N = 15,271**  **Cases = 3,314** | | **Evening activity Q3**  **N = 15,274**  **Cases = 2,565** | | **Evening activity Q4**  **N = 15,288**  **Cases = 1,745** | |
| --- | --- | --- | --- | --- | --- | --- | --- | --- |
| **Morning activity** | **OR** | **95% CI** | **OR** | **95% CI** | **OR** | **95% CI** | **OR** | **95% CI** |
| 1^st^ Quantile | 1.00 | - | 1.00 | - | 1.00 | - | 1.00 | - |
| 2^nd^ Quantile | 0.83 | 0.76, 0.92 | 0.88 | 0.79, 0.98 | 0.82 | 0.73, 0.93 | 0.79 | 0.68, 0.92 |
| 3^rd^ Quantile | 0.77 | 0.69, 0.85 | 0.85 | 0.76, 0.96 | 0.90 | 0.79, 1.02 | 0.84 | 0.72, 0.98 |
| 4^th^ Quantile | 0.60 | 0.53, 0.68 | 0.78 | 0.69, 0.89 | 0.75 | 0.65, 0.86 | 0.77 | 0.66, 0.90 |
| OR: Odds ratio, CI: Confidence interval  Models adjusted for time period, sex, age, study region, education, employment status, risky alcohol status, smoking, night shift status, and sleep duration. | | | | | | | | |

# Odds ratios and 95% confidence intervals for morning activity in relation to diabetes, stratified by evening activity

| **Physical activity timing** | **Evening activity Q1**  **N = 15,283**  **Cases = 1,431** | | **Evening activity Q2**  **N = 15,271**  **Cases = 1,031** | | **Evening activity Q3**  **N = 15,274**  **Cases = 752** | | **Evening activity Q4**  **N = 15,288**  **Cases = 573** | |
| --- | --- | --- | --- | --- | --- | --- | --- | --- |
| **Morning activity** | **OR** | **95% CI** | **OR** | **95% CI** | **OR** | **95% CI** | **OR** | **95% CI** |
| 1^st^ Quantile | 1.00 | - | 1.00 | - | 1.00 | - | 1.00 | - |
| 2^nd^ Quantile | 0.84 | 0.73, 0.97 | 0.92 | 0.77, 1.10 | 0.92 | 0.74, 1.14 | 0.76 | 0.58, 1.00 |
| 3^rd^ Quantile | 0.72 | 0.60, 0.85 | 0.98 | 0.81, 1.19 | 1.00 | 0.80, 1.24 | 0.89 | 0.69, 1.15 |
| 4^th^ Quantile | 0.61 | 0.50, 0.75 | 0.94 | 0.76, 1.17 | 0.94 | 0.74, 1.19 | 0.82 | 0.63, 1.06 |
| OR: Odds ratio, CI: Confidence interval  Models adjusted for time period, sex, age, study region, education, employment status, risky alcohol status, smoking, night shift status, and sleep duration. | | | | | | | | |

# Odds ratios and 95% confidence intervals for morning and evening activity in relation to diabetes, stratified by afternoon activity

| **Physical activity timing** | **Afternoon Q1**  **N = 15,283**  **Cases = 1,529** | | **Afternoon Q2**  **N = 15,271**  **Cases = 925** | | **Afternoon Q3**  **N = 15,274**  **Cases = 785** | | **Afternoon Q4**  **N = 15,288**  **Cases = 548** | |
| --- | --- | --- | --- | --- | --- | --- | --- | --- |
| **Morning** | **OR** | **95% CI** | **OR** | **95% CI** | **OR** | **95% CI** | **OR** | **95% CI** |
| 1^st^ Quantile | 1.00 | - | 1.00 | - | 1.00 | - | 1.00 | - |
| 2^nd^ Quantile | 0.80 | 0.70, 0.91 | 1.03 | 0.85, 1.23 | 1.00 | 0.78, 1.29 | 0.80 | 0.56, 1.15 |
| 3^rd^ Quantile | 0.67 | 0.56, 0.81 | 1.05 | 0.87, 1.27 | 1.14 | 0.90, 1.45 | 0.91 | 0.66, 1.25 |
| 4^th^ Quantile | 0.48 | 0.36, 0.62 | 0.84 | 0.67, 1.06 | 1.04 | 0.82, 1.33 | 1.00 | 0.74, 1.34 |
| **Evening** |  |  |  |  |  |  |  |  |
| 1^st^ Quantile | 1.00 | - | 1.00 | - | 1.00 | - | 1.00 | - |
| 2^nd^ Quantile | 0.74 | 0.64, 0.84 | 0.90 | 0.76, 1.08 | 1.08 | 0.87, 1.34 | 0.92 | 0.67, 1.25 |
| 3^rd^ Quantile | 0.53 | 0.44, 0.64 | 0.73 | 0.61, 0.88 | 0.84 | 0.67, 1.04 | 0.87 | 0.64, 1.16 |
| 4^th^ Quantile | 0.33 | 0.25, 0.44 | 0.66 | 0.53, 0.82 | 0.65 | 0.52, 0.83 | 0.81 | 0.61, 1.08 |
| OR: Odds ratio, CI: Confidence interval  Models adjusted for time period, sex, age, study region, education, employment status, risky alcohol status, smoking, night shift status, and sleep duration. | | | | | | | | |

# Re-allocating physical activity from one daytime period to another

| **Physical activity timing** | **Obesity** | | **Diabetes** | |
| --- | --- | --- | --- | --- |
|  | Odds ratio | 95% CI | Odds ratio | 95% CI |
| Replacing Morning activity with |  |  |  |  |
| *Afternoon activity* | 0.77 | 0.74, 0.80 | 0.85 | 0.79, 0.90 |
| *Evening activity* | 0.77 | 0.74, 0.80 | 0.81 | 0.76, 0.87 |
| Replacing Afternoon activity with |  |  |  |  |
| *Morning activity* | 1.47 | 1.31, 1.65 | 1.39 | 1.16, 1.66 |
| *Evening activity* | 1.01 | 0.96, 1.06 | 0.97 | 0.89, 1.05 |
| Replacing Evening activity with |  |  |  |  |
| *Morning activity* | 1.46 | 1.30, 1.63 | 1.44 | 1.20, 1.71 |
| *Afternoon activity* | 0.99 | 0.94, 1.04 | 1.03 | 0.95, 1.12 |
| CI: Confidence interval.  Adjusted for sex, age, study region, education, employment status, alcohol use, smoking status, night shift work frequency, sleep duration, total daytime activity, and physical activity in the respective two time periods. | | | | |

# Timing of physical activity in relation to obesity and diabetes with shifts in time period definitions

| **Obesity** | | | | | | |
| --- | --- | --- | --- | --- | --- | --- |
| **Physical activity timing** | **Time periods 1 hour earlier** | | | **Time periods 1 hour later** | | |
|  | Cases | Odds ratio | 95% CI | Cases | Odds ratio | 95% CI |
| **Morning** |  |  |  |  |  |  |
| 1^st^ Quartile | 3,854 | 1.00 | - | 4,283 | 1.00 | - |
| 2^nd^ Quartile | 3,145 | 0.92 | 0.87, 0.98 | 3,193 | 0.79 | 0.74, 0.83 |
| 3^rd^ Quartile | 2,763 | 0.91 | 0.86, 0.97 | 2,604 | 0.68 | 0.64, 0.73 |
| 4^th^ Quartile | 2,360 | 0.87 | 0.82, 0.93 | 2,042 | 0.59 | 0.55, 0.63 |
| **Afternoon** |  |  |  |  |  |  |
| 1^st^ Quartile | 4,698 | 1.00 | - | 4,882 | 1.00 | - |
| 2^nd^ Quartile | 3,185 | 0.69 | 0.65, 0.73 | 3,215 | 0.67 | 0.64, 0.71 |
| 3^rd^ Quartile | 2,544 | 0.57 | 0.53, 0.60 | 2,465 | 0.54 | 0.50, 0.57 |
| 4^th^ Quartile | 1,695 | 0.38 | 0.36, 0.41 | 1,560 | 0.35 | 0.32, 0.37 |
| **Evening** |  |  |  |  |  |  |
| 1^st^ Quartile | 4,664 | 1.00 | - | 4,304 | 1.00 | - |
| 2^nd^ Quartile | 3,352 | 0.74 | 0.70, 0.78 | 3,278 | 0.85 | 0.80, 0.89 |
| 3^rd^ Quartile | 2,429 | 0.54 | 0.51, 0.58 | 2,653 | 0.74 | 0.70, 0.79 |
| 4^th^ Quartile | 1,677 | 0.40 | 0.37, 0.43 | 1,887 | 0.56 | 0.53, 0.60 |
| **Nighttime** |  |  |  |  |  |  |
| 1^st^ Quartile | 2,997 | 1.00 | - | 2,728 | 1.00 | - |
| 2^nd^ Quartile | 3,124 | 1.14 | 1.07, 1.21 | 2,966 | 1.19 | 1.12, 1.27 |
| 3^rd^ Quartile | 3,197 | 1.27 | 1.20, 1.35 | 3,201 | 1.38 | 1.30, 1.46 |
| 4^th^ Quartile | 2,804 | 1.22 | 1.14, 1.30 | 3,227 | 1.47 | 1.37, 1.56 |
|  | | | | | | |
| **Diabetes** | | | | | | |
| **Physical activity timing** | **Time periods 1 hour earlier** | | | **Time periods 1 hour later** | | |
|  | Cases | Odds ratio | 95% CI | Cases | Odds ratio | 95% CI |
| **Morning** |  |  |  |  |  |  |
| 1^st^ Quartile | 1,218 | 1.00 | - | 1,337 | 1.00 | - |
| 2^nd^ Quartile | 970 | 0.96 | 0.87, 1.05 | 988 | 0.84 | 0.77, 0.92 |
| 3^rd^ Quartile | 847 | 0.94 | 0.85, 1.04 | 804 | 0.75 | 0.67, 0.82 |
| 4^th^ Quartile | 752 | 0.93 | 0.84, 1.04 | 658 | 0.67 | 0.60, 0.75 |
| **Afternoon** |  |  |  |  |  |  |
| 1^st^ Quartile | 1,454 | 1.00 | - | 1,540 | 1.00 | - |
| 2^nd^ Quartile | 1,000 | 0.79 | 0.72, 0.86 | 944 | 0.71 | 0.65, 0.78 |
| 3^rd^ Quartile | 764 | 0.64 | 0.57, 0.70 | 782 | 0.65 | 0.58, 0.72 |
| 4^th^ Quartile | 569 | 0.50 | 0.44, 0.56 | 521 | 0.46 | 0.40, 0.52 |
| **Evening** |  |  |  |  |  |  |
| 1^st^ Quartile | 1,493 | 1.00 | - | 1,371 | 1.00 | - |
| 2^nd^ Quartile | 988 | 0.76 | 0.69, 0.83 | 1,016 | 0.87 | 0.79, 0.95 |
| 3^rd^ Quartile | 740 | 0.61 | 0.55, 0.68 | 792 | 0.76 | 0.69, 0.84 |
| 4^th^ Quartile | 566 | 0.52 | 0.46, 0.58 | 608 | 0.65 | 0.58, 0.73 |
| **Nighttime** |  |  |  |  |  |  |
| 1^st^ Quartile | 932 | 1.00 | - | 868 | 1.00 | - |
| 2^nd^ Quartile | 993 | 1.13 | 1.03, 1.24 | 997 | 1.25 | 1.13, 1.38 |
| 3^rd^ Quartile | 1,001 | 1.22 | 1.10, 1.34 | 963 | 1.29 | 1.17, 1.43 |
| 4^th^ Quartile | 861 | 1.16 | 1.04, 1.29 | 959 | 1.39 | 1.25, 1.54 |
| CI: Confidence interval.  Models adjusted for sex, age, study region, education, employment status, alcohol use, smoking status, night shift work frequency, and sleep duration.  Time periods 1 hour earlier: Morning (05:00 to 10:59), afternoon (11:00 to 16:59), evening (17:00 to 22:59), and night (23:00 to 04:59).  Time periods 1 hour later: Morning (07:00 to 12:59), afternoon (13:00 to 18:59), evening (19:00 to 00:59), and night (01:00 to 06:59). | | | | | | |

# Timing of physical activity in relation to obesity, stratified by employment status

| **Physical activity timing** | **Employed**  **N = 45,734** | | | **Unemployed**  **N = 1,506** | | | | | **Outside the labor force**  **N = 11,248** | | |
| --- | --- | --- | --- | --- | --- | --- | --- | --- | --- | --- | --- |
|  | **Cases** | **Odds ratio** | **95% CI** | | **Cases** | **Odds ratio** | **95% CI** | **Cases** | | **Odds ratio** | **95% CI** |
| **Morning** |  |  |  | |  |  |  |  | |  |  |
| 1^st^ Quantile | 2,602 | 1.00 | - | | 241 | 1.00 | - | 1,208 | | 1.00 | - |
| 2^nd^ Quantile | 2,179 | 0.82 | 0.77, 0.88 | | 113 | 1.18 | 0.87, 1.60 | 781 | | 0.83 | 0.74, 0.93 |
| 3^rd^ Quantile | 2,017 | 0.83 | 0.77, 0.89 | | 75 | 0.91 | 0.64, 1.29 | 651 | | 0.81 | 0.71, 0.92 |
| 4^th^ Quantile | 1,652 | 0.73 | 0.67, 0.79 | | 48 | 0.75 | 0.50, 1.14 | 477 | | 0.64 | 0.55, 0.73 |
| **Afternoon** |  |  |  | |  |  |  |  | |  |  |
| 1^st^ Quantile | 3,068 | 1.00 | - | | 279 | 1.00 | - | 1,443 | | 1.00 | - |
| 2^nd^ Quantile | 2,277 | 0.69 | 0.65, 0.74 | | 94 | 0.58 | 0.42, 0.79 | 800 | | 0.63 | 0.56, 0.71 |
| 3^rd^ Quantile | 1,868 | 0.57 | 0.53, 0.62 | | 57 | 0.44 | 0.30, 0.64 | 563 | | 0.47 | 0.41, 0.54 |
| 4^th^ Quantile | 1,237 | 0.39 | 0.36, 0.43 | | 47 | 0.34 | 0.22, 0.51 | 311 | | 0.25 | 0.21, 0.29 |
| **Evening** |  |  |  | |  |  |  |  | |  |  |
| 1^st^ Quantile | 2,863 | 1.00 | - | | 253 | 1.00 | - | 1,353 | | 1.00 | - |
| 2^nd^ Quantile | 2,314 | 0.80 | 0.75, 0.86 | | 113 | 0.66 | 0.49, 0.89 | 859 | | 0.75 | 0.67, 0.84 |
| 3^rd^ Quantile | 1,919 | 0.65 | 0.61, 0.70 | | 69 | 0.52 | 0.36, 0.74 | 569 | | 0.60 | 0.53, 0.68 |
| 4^th^ Quantile | 1,354 | 0.46 | 0.42, 0.49 | | 42 | 0.37 | 0.24, 0.57 | 336 | | 0.45 | 0.38, 0.52 |
| **Night** |  |  |  | |  |  |  |  | |  |  |
| 1^st^ Quantile | 1,765 | 1.00 | - | | 107 | 1.00 | - | 729 | | 1.00 | - |
| 2^nd^ Quantile | 1,984 | 1.22 | 1.13, 1.31 | | 119 | 1.04 | 0.74, 1.46 | 823 | | 1.36 | 1.21, 1.54 |
| 3^rd^ Quantile | 2,259 | 1.43 | 1.33, 1.54 | | 147 | 1.44 | 1.03, 2.01 | 831 | | 1.57 | 1.39, 1.78 |
| 4^th^ Quantile | 2,442 | 1.50 | 1.39, 1.62 | | 104 | 1.72 | 1.18, 2.51 | 734 | | 1.81 | 1.58, 2.08 |
| OR: Odds ratio, CI: Confidence interval.  “Outside the labor force” refers to individuals who are not currently working but who are not unemployed.  Models adjusted for time period, sex, age, study region, education, risky alcohol status, smoking, night shift status, and sleep duration. | | | | | | | | | | | |

# Timing of physical activity in relation to diabetes, stratified by employment status

| **Physical activity timing** | **Employed**  **N = 47,423** | | | **Unemployed**  **N = 1,598** | | | | | **Outside the labor force**  **N = 11,703** | | |
| --- | --- | --- | --- | --- | --- | --- | --- | --- | --- | --- | --- |
|  | **Cases** | **Odds ratio** | **95% CI** | | **Cases** | **Odds ratio** | **95% CI** | **Cases** | | **Odds ratio** | **95% CI** |
| **Morning** |  |  |  | |  |  |  |  | |  |  |
| 1^st^ Quantile | 610 | 1.00 | - | | 98 | 1.00 | - | 570 | | 1.00 | - |
| 2^nd^ Quantile | 494 | 0.81 | 0.72, 0.92 | | 46 | 1.09 | 0.72, 1.66 | 391 | | 0.92 | 0.80, 1.07 |
| 3^rd^ Quantile | 499 | 0.88 | 0.77, 1.00 | | 21 | 0.57 | 0.33, 0.99 | 322 | | 0.89 | 0.75, 1.04 |
| 4^th^ Quantile | 446 | 0.83 | 0.72, 0.96 | | 14 | 0.63 | 0.33, 1.21 | 253 | | 0.76 | 0.64, 0.92 |
| **Afternoon** |  |  |  | |  |  |  |  | |  |  |
| 1^st^ Quantile | 692 | 1.00 | - | | 105 | 1.00 | - | 719 | | 1.00 | - |
| 2^nd^ Quantile | 527 | 0.76 | 0.68, 0.87 | | 39 | 0.96 | 0.62, 1.50 | 355 | | 0.61 | 0.52, 0.71 |
| 3^rd^ Quantile | 476 | 0.71 | 0.62, 0.81 | | 23 | 0.58 | 0.32, 1.05 | 286 | | 0.55 | 0.47, 0.66 |
| 4^th^ Quantile | 354 | 0.56 | 0.48, 0.66 | | 16 | 0.51 | 0.26, 0.96 | 176 | | 0.35 | 0.29, 0.43 |
| **Evening** |  |  |  | |  |  |  |  | |  |  |
| 1^st^ Quantile | 661 | 1.00 | - | | 102 | 1.00 | - | 656 | | 1.00 | - |
| 2^nd^ Quantile | 554 | 0.86 | 0.77, 0.97 | | 38 | 0.58 | 0.37, 0.89 | 432 | | 0.85 | 0.74, 0.98 |
| 3^rd^ Quantile | 459 | 0.71 | 0.62, 0.80 | | 23 | 0.48 | 0.27, 0.83 | 268 | | 0.66 | 0.55, 0.78 |
| 4^th^ Quantile | 375 | 0.57 | 0.49, 0.66 | | 16 | 0.48 | 0.25, 0.93 | 180 | | 0.57 | 0.46, 0.70 |
| **Night** |  |  |  | |  |  |  |  | |  |  |
| 1^st^ Quantile | 433 | 1.00 | - | | 44 | 1.00 | - | 365 | | 1.00 | - |
| 2^nd^ Quantile | 509 | 1.22 | 1.06, 1.39 | | 49 | 1.10 | 0.69, 1.76 | 386 | | 1.17 | 1.00, 1.37 |
| 3^rd^ Quantile | 499 | 1.17 | 1.02, 1.34 | | 54 | 1.21 | 0.76, 1.93 | 405 | | 1.39 | 1.18, 1.63 |
| 4^th^ Quantile | 608 | 1.33 | 1.16, 1.52 | | 32 | 1.07 | 0.62, 1.85 | 380 | | 1.63 | 1.38, 1.93 |
| OR: Odds ratio, CI: Confidence interval.  “Outside the labor force” refers to individuals who are not currently working but who are not unemployed.  Models adjusted for time period, sex, age, study region, education, risky alcohol status, smoking, night shift status, and sleep duration. | | | | | | | | | | | |

# Timing of physical activity in relation to obesity and diabetes among non-night shift workers

| **Physical activity timing** | **Obesity**  **N=35,506** | | **Diabetes**  **N=35,506** | |
| --- | --- | --- | --- | --- |
|  | **Odds ratio** | **95% confidence interval** | **Odds ratio** | **95% confidence interval** |
| **Morning** |  |  |  |  |
| 1^st^ Quartile | 1.00 | - | 1.00 | - |
| 2^nd^ Quartile | 0.84 | 0.78, 0.91 | 0.78 | 0.67, 0.92 |
| 3^rd^ Quartile | 0.85 | 0.78, 0.92 | 0.90 | 0.77, 1.06 |
| 4^th^ Quartile | 0.76 | 0.70, 0.84 | 0.80 | 0.67, 0.95 |
| **Afternoon** | 0.84 | 0.78, 0.91 |  |  |
| 1^st^ Quartile | 1.00 | - | 1.00 | - |
| 2^nd^ Quartile | 0.69 | 0.64, 0.75 | 0.74 | 0.64, 0.86 |
| 3^rd^ Quartile | 0.58 | 0.53, 0.63 | 0.70 | 0.60, 0.83 |
| 4^th^ Quartile | 0.37 | 0.34, 0.41 | 0.55 | 0.45, 0.67 |
| **Evening** |  |  |  |  |
| 1^st^ Quartile | 1.00 | - | 1.00 | - |
| 2^nd^ Quartile | 0.81 | 0.75, 0.87 | 0.80 | 0.69, 0.92 |
| 3^rd^ Quartile | 0.65 | 0.60, 0.70 | 0.68 | 0.58, 0.80 |
| 4^th^ Quartile | 0.44 | 0.40, 0.48 | 0.57 | 0.48, 0.68 |
| **Nighttime** |  |  |  |  |
| 1^st^ Quartile | 1.00 | - | 1.00 | - |
| 2^nd^ Quartile | 1.22 | 1.12, 1.32 | 1.26 | 1.07, 1.48 |
| 3^rd^ Quartile | 1.47 | 1.35, 1.60 | 1.22 | 1.04, 1.44 |
| 4^th^ Quartile | 1.53 | 1.40, 1.67 | 1.39 | 1.18, 1.65 |
| Models adjusted for time period, sex, age, study region, education, employment status, risky alcohol status, smoking, and sleep duration. | | | | |

# Timing of physical activity in relation to obesity and diabetes stratified by sex

| **Obesity** | | | | |
| --- | --- | --- | --- | --- |
| **Physical activity timing** | **Men**  **N=29,407** | | **Women**  **N=31,709** | |
|  | **Odds ratio** | **95% confidence interval** | **Odds ratio** | **95% confidence interval** |
| **Morning** |  |  |  |  |
| 1^st^ Quartile | 1.00 | - | 1.00 | - |
| 2^nd^ Quartile | 0.88 | 0.81, 0.95 | 0.79 | 0.73, 0.86 |
| 3^rd^ Quartile | 0.93 | 0.85, 1.01 | 0.74 | 0.68, 0.80 |
| 4^th^ Quartile | 0.82 | 0.74, 0.90 | 0.61 | 0.55, 0.67 |
| **Afternoon** |  |  |  |  |
| 1^st^ Quartile | 1.00 | - | 1.00 | - |
| 2^nd^ Quartile | 0.69 | 0.64, 0.75 | 0.65 | 0.60, 0.70 |
| 3^rd^ Quartile | 0.57 | 0.52, 0.63 | 0.51 | 0.47, 0.56 |
| 4^th^ Quartile | 0.38 | 0.35, 0.42 | 0.33 | 0.30, 0.36 |
| **Evening** |  |  |  |  |
| 1^st^ Quartile | 1.00 | - | 1.00 | - |
| 2^nd^ Quartile | 0.80 | 0.74, 0.86 | 0.78 | 0.72, 0.84 |
| 3^rd^ Quartile | 0.64 | 0.59, 0.69 | 0.63 | 0.58, 0.69 |
| 4^th^ Quartile | 0.47 | 0.42, 0.51 | 0.44 | 0.40, 0.49 |
| **Nighttime** |  |  |  |  |
| 1^st^ Quartile | 1.00 | - | 1.00 | - |
| 2^nd^ Quartile | 1.14 | 1.04, 1.24 | 1.35 | 1.24, 1.48 |
| 3^rd^ Quartile | 1.34 | 1.23, 1.46 | 1.60 | 1.47, 1.74 |
| 4^th^ Quartile | 1.43 | 1.30, 1.56 | 1.74 | 1.59, 1.90 |
|  | | | | |
| **Diabetes** | | | | |
| **Morning** |  |  |  |  |
| 1^st^ Quartile | 1.00 | - | 1.00 | - |
| 2^nd^ Quartile | 0.87 | 0.77, 0.99 | 0.84 | 0.73, 0.96 |
| 3^rd^ Quartile | 0.86 | 0.75, 0.98 | 0.87 | 0.75, 1.00 |
| 4^th^ Quartile | 0.80 | 0.69, 0.93 | 0.78 | 0.67, 0.92 |
| **Afternoon** |  |  |  |  |
| 1^st^ Quartile | 1.00 | - | 1.00 | - |
| 2^nd^ Quartile | 0.68 | 0.60, 0.77 | 0.72 | 0.63, 0.82 |
| 3^rd^ Quartile | 0.60 | 0.52, 0.69 | 0.68 | 0.58, 0.79 |
| 4^th^ Quartile | 0.49 | 0.42, 0.58 | 0.44 | 0.37, 0.53 |
| **Evening** |  |  |  |  |
| 1^st^ Quartile | 1.00 | - | 1.00 | - |
| 2^nd^ Quartile | 0.86 | 0.76, 0.97 | 0.80 | 0.70, 0.91 |
| 3^rd^ Quartile | 0.66 | 0.57, 0.75 | 0.67 | 0.58, 0.78 |
| 4^th^ Quartile | 0.53 | 0.45, 0.62 | 0.59 | 0.50, 0.70 |
| **Nighttime** |  |  |  |  |
| 1^st^ Quartile | 1.00 | - | 1.00 | - |
| 2^nd^ Quartile | 1.15 | 1.00, 1.31 | 1.26 | 1.09, 1.46 |
| 3^rd^ Quartile | 1.18 | 1.03, 1.36 | 1.34 | 1.16, 1.55 |
| 4^th^ Quartile | 1.41 | 1.22, 1.62 | 1.43 | 1.22, 1.66 |
| Models adjusted for time period, age, study region, education, employment status, risky alcohol status, smoking, night shift status, and sleep duration. | | | | |

# Timing of physical activity in relation to diabetes with additional adjustment for body mass index

| **Physical activity timing** | **Odds ratio** | **95% confidence interval** |
| --- | --- | --- |
| **Morning** |  |  |
| 1^st^ Quartile | 1.00 | - |
| 2^nd^ Quartile | 0.91 | 0.83, 1.00 |
| 3^rd^ Quartile | 0.94 | 0.85, 1.04 |
| 4^th^ Quartile | 0.92 | 0.82, 1.02 |
| **Afternoon** |  |  |
| 1^st^ Quartile | 1.00 | - |
| 2^nd^ Quartile | 0.82 | 0.74, 0.90 |
| 3^rd^ Quartile | 0.80 | 0.72, 0.88 |
| 4^th^ Quartile | 0.64 | 0.57, 0.73 |
| **Evening** |  |  |
| 1^st^ Quartile | 1.00 | - |
| 2^nd^ Quartile | 0.91 | 0.83, 1.00 |
| 3^rd^ Quartile | 0.78 | 0.70, 0.86 |
| 4^th^ Quartile | 0.70 | 0.63, 0.79 |
| **Nighttime** |  |  |
| 1^st^ Quartile | 1.00 | - |
| 2^nd^ Quartile | 1.11 | 1.01, 1.23 |
| 3^rd^ Quartile | 1.10 | 0.99, 1.22 |
| 4^th^ Quartile | 1.21 | 1.09, 1.34 |
| Models adjusted for time period, sex, age, study region, education, employment status, risky alcohol status, smoking, night shift status, sleep duration, and body mass index. | | |

# Linear regression models for physical activity timing in relation to body mass index and HbA1c levels

| **Physical activity timing** | **Body mass index**  **N=61,116** | | **HbA1c**  **N=58,756** | |
| --- | --- | --- | --- | --- |
|  | **ß** | **95% confidence interval** | **ß** | **95% confidence interval** |
| **Morning** |  |  |  |  |
| 1^st^ Quartile | 0.00 | - | 0.00 | - |
| 2^nd^ Quartile | -0.43 | -0.53, -0.33 | -0.18 | -0.32, -0.05 |
| 3^rd^ Quartile | -0.45 | -0.55, -0.34 | -0.18 | -0.32, -0.05 |
| 4^th^ Quartile | -0.73 | -0.84, -0.62 | -0.17 | -0.31, -0.02 |
| **Afternoon** |  |  |  |  |
| 1^st^ Quartile | 0.00 | - | 0.00 | - |
| 2^nd^ Quartile | -1.04 | -1.14, -0.94 | -0.55 | -0.68, -0.41 |
| 3^rd^ Quartile | -1.41 | -1.52, -1.30 | -0.66 | -0.80, -0.52 |
| 4^th^ Quartile | -2.09 | -2.21, -1.98 | -0.95 | -1.10, -0.80 |
| **Evening** |  |  |  |  |
| 1^st^ Quartile | 0.00 | - | 0.00 | - |
| 2^nd^ Quartile | -0.66 | -0.76, -0.56 | -0.18 | -0.31, -0.05 |
| 3^rd^ Quartile | -1.13 | -1.23, -1.03 | -0.30 | -0.43, -0.16 |
| 4^th^ Quartile | -1.65 | -1.75, -1.54 | -0.40 | -0.54, -0.26 |
| **Nighttime** |  |  |  |  |
| 1^st^ Quartile | 0.00 | - | 0.00 | - |
| 2^nd^ Quartile | 0.35 | 0.26, 0.45 | 0.25 | 0.12, 0.38 |
| 3^rd^ Quartile | 0.68 | 0.58, 0.78 | 0.31 | 0.18, 0.45 |
| 4^th^ Quartile | 0.84 | 0.73, 0.94 | 0.60 | 0.47, 0.74 |
| Models adjusted for time period, sex, age, and study region, education, employment status, risky alcohol status, smoking, night shift status, and sleep duration. | | | | |

# Timing of physical activity in relation to diabetes (self-reported and based on HbA1c levels) when restricting the analyses to centrally analyzed HbA1c measurements

| **Physical activity timing** | **Cases** | **Odds ratio** | **95% CI** |
| --- | --- | --- | --- |
| **Morning** |  |  |  |
| 1^st^ Quartile | 1,206 | 1.00 | - |
| 2^nd^ Quartile | 866 | 0.85 | 0.77, 0.93 |
| 3^rd^ Quartile | 803 | 0.88 | 0.80, 0.98 |
| 4^th^ Quartile | 668 | 0.80 | 0.71, 0.89 |
| **Afternoon** |  |  |  |
| 1^st^ Quartile | 1,432 | 1.00 | - |
| 2^nd^ Quartile | 861 | 0.69 | 0.63, 0.76 |
| 3^rd^ Quartile | 739 | 0.64 | 0.58, 0.72 |
| 4^th^ Quartile | 511 | 0.47 | 0.42, 0.53 |
| **Evening** |  |  |  |
| 1^st^ Quartile | 1,348 | 1.00 | - |
| 2^nd^ Quartile | 959 | 0.82 | 0.75, 0.90 |
| 3^rd^ Quartile | 710 | 0.67 | 0.61, 0.74 |
| 4^th^ Quartile | 526 | 0.55 | 0.49, 0.62 |
| **Night** |  |  |  |
| 1^st^ Quartile | 781 | 1.00 | - |
| 2^nd^ Quartile | 899 | 1.24 | 1.12, 1.37 |
| 3^rd^ Quartile | 927 | 1.32 | 1.19, 1.47 |
| 4^th^ Quartile | 936 | 1.43 | 1.28, 1.59 |
| CI: Confidence interval.  Models adjusted for time period, sex, age, study region, education, employment status, risky alcohol status, smoking, night shift status, and sleep duration. | | | |

# Timing of physical activity in relation to obesity and diabetes with additional adjustment for average measurement hours

| **Physical activity timing** | **Obesity**  **N=61,116** | | **Diabetes**  **N=61,116** | |
| --- | --- | --- | --- | --- |
|  | **Odds ratio** | **95% confidence interval** | **Odds ratio** | **95% confidence interval** |
| **Morning** |  |  |  |  |
| 1^st^ Quartile | 1.00 | - | 1.00 | - |
| 2^nd^ Quartile | 0.83 | 0.78, 0.87 | 0.85 | 0.77, 0.93 |
| 3^rd^ Quartile | 0.82 | 0.77, 0.87 | 0.85 | 0.77, 0.94 |
| 4^th^ Quartile | 0.70 | 0.65, 0.75 | 0.78 | 0.70, 0.87 |
| **Afternoon** |  |  |  |  |
| 1^st^ Quartile | 1.00 | - | 1.00 | - |
| 2^nd^ Quartile | 0.67 | 0.63, 0.71 | 0.69 | 0.63, 0.76 |
| 3^rd^ Quartile | 0.54 | 0.51, 0.58 | 0.63 | 0.57, 0.70 |
| 4^th^ Quartile | 0.36 | 0.33, 0.38 | 0.46 | 0.41, 0.52 |
| **Evening** |  |  |  |  |
| 1^st^ Quartile | 1.00 | - | 1.00 | - |
| 2^nd^ Quartile | 0.78 | 0.74, 0.83 | 0.83 | 0.76, 0.91 |
| 3^rd^ Quartile | 0.63 | 0.59, 0.67 | 0.66 | 0.60, 0.73 |
| 4^th^ Quartile | 0.45 | 0.42, 0.48 | 0.55 | 0.49, 0.62 |
| **Nighttime** |  |  |  |  |
| 1^st^ Quartile | 1.00 | - | 1.00 | - |
| 2^nd^ Quartile | 1.25 | 1.18, 1.33 | 1.20 | 1.09, 1.33 |
| 3^rd^ Quartile | 1.47 | 1.39, 1.57 | 1.26 | 1.14, 1.39 |
| 4^th^ Quartile | 1.57 | 1.48, 1.68 | 1.41 | 1.28, 1.57 |
| Models adjusted for time period, sex, age, study region, education, employment status, risky alcohol status, smoking, sleep duration, and average measurement hours. | | | | |

# Timing of physical activity in relation to obesity and diabetes with additional adjustment for midpoint of sleep

| **Physical activity timing** | **Obesity**  **N=** **54,391** | | **Diabetes**  **N=54,391** | |
| --- | --- | --- | --- | --- |
|  | **Odds ratio** | **95% confidence interval** | **Odds ratio** | **95% confidence interval** |
| **Morning** |  |  |  |  |
| 1^st^ Quartile | 1.00 | - | 1.00 | - |
| 2^nd^ Quartile | 0.80 | 0.75, 0.85 | 0.84 | 0.75, 0.93 |
| 3^rd^ Quartile | 0.78 | 0.73, 0.84 | 0.85 | 0.76, 0.95 |
| 4^th^ Quartile | 0.68 | 0.63, 0.74 | 0.76 | 0.67, 0.87 |
| **Afternoon** |  |  |  |  |
| 1^st^ Quartile | 1.00 | - | 1.00 | - |
| 2^nd^ Quartile | 0.67 | 0.63, 0.71 | 0.69 | 0.62, 0.77 |
| 3^rd^ Quartile | 0.54 | 0.50, 0.58 | 0.63 | 0.57, 0.71 |
| 4^th^ Quartile | 0.35 | 0.33, 0.38 | 0.46 | 0.41, 0.53 |
| **Evening** |  |  |  |  |
| 1^st^ Quartile | 1.00 | - | 1.00 | - |
| 2^nd^ Quartile | 0.80 | 0.75, 0.84 | 0.81 | 0.74, 0.90 |
| 3^rd^ Quartile | 0.64 | 0.60, 0.68 | 0.68 | 0.61, 0.76 |
| 4^th^ Quartile | 0.46 | 0.42, 0.49 | 0.57 | 0.50, 0.65 |
| **Nighttime** |  |  |  |  |
| 1^st^ Quartile | 1.00 | - | 1.00 | - |
| 2^nd^ Quartile | 1.24 | 1.16, 1.32 | 1.21 | 1.08, 1.35 |
| 3^rd^ Quartile | 1.46 | 1.37, 1.56 | 1.25 | 1.12, 1.40 |
| 4^th^ Quartile | 1.54 | 1.44, 1.65 | 1.43 | 1.27, 1.60 |
| Models adjusted for time period, sex, age, study region, education, employment status, risky alcohol status, smoking, sleep duration, and midpoint of sleep. | | | | |
